# Evaluating the effects of delivering integrated kinesthetic and tactile cues to individuals with unilateral hemiparetic stroke during overground walking

**DOI:** 10.1186/s12984-018-0372-0

**Published:** 2018-04-16

**Authors:** Muhammad Raheel Afzal, Sanghun Pyo, Min-Kyun Oh, Young Sook Park, Jungwon Yoon

**Affiliations:** 1 School of Integrated Technology, Gwangju Institute of Science and Technology, 123 Cheomdangwagi-ro, Buk-gu, Gwangju, 61005 Republic of Korea; 2 Department of Rehabilitation Medicine, Gyeongsang National University School of Medicine, Gyeongsang National University Hospital, Jinju, 52727 Republic of Korea; 3 Department of Physical Medicine and Rehabilitation, Sungkyunkwan University School of Medicine, Samsung Changwon Hospital, Changwon, 51353 Republic of Korea

**Keywords:** Haptics, Gait rehabilitation, Stroke, Symmetry, Trunk sway, Muscle activity

## Abstract

**Background:**

Integration of kinesthetic and tactile cues for application to post-stroke gait rehabilitation is a novel concept which needs to be explored. The combined provision of haptic cues may result in collective improvement of gait parameters such as symmetry, balance and muscle activation patterns. Our proposed integrated cue system can offer a cost-effective and voluntary gait training experience for rehabilitation of subjects with unilateral hemiparetic stroke.

**Methods:**

Ten post-stroke ambulatory subjects participated in a 10 m walking trial while utilizing the haptic cues (either alone or integrated application), at their preferred and increased gait speeds. In the system a haptic cane device (HCD) provided kinesthetic perception and a vibrotactile feedback device (VFD) provided tactile cue on the paretic leg for gait modification. Balance, gait symmetry and muscle activity were analyzed to identify the benefits of utilizing the proposed system.

**Results:**

When using kinesthetic cues, either alone or integrated with a tactile cue, an increase in the percentage of non-paretic peak activity in the paretic muscles was observed at the preferred gait speed (vastus medialis obliquus: *p* <  0.001, partial eta squared (η^2^) = 0.954; semitendinosus p <  0.001, partial η^2^ = 0.793) and increased gait speeds (vastus medialis obliquus: p <  0.001, partial η^2^ = 0.881; semitendinosus *p* = 0.028, partial η^2^ = 0.399). While using HCD and VFD (individual and integrated applications), subjects could walk at their preferred and increased gait speeds without disrupting trunk balance in the mediolateral direction. The temporal stance symmetry ratio was improved when using tactile cues, either alone or integrated with a kinesthetic cue, at their preferred gait speed (*p* <  0.001, partial η^2^ = 0.702).

**Conclusions:**

When combining haptic cues, the subjects walked at their preferred gait speed with increased temporal stance symmetry and paretic muscle activity affecting their balance. Similar improvements were observed at higher gait speeds. The efficacy of the proposed system is influenced by gait speed. Improvements were observed at a 20% increased gait speed, whereas, a plateau effect was observed at a 40% increased gait speed. These results imply that integration of haptic cues may benefit post-stroke gait rehabilitation by inducing simultaneous improvements in gait symmetry and muscle activity.

## Background

Worldwide, stroke is ranked as one of the leading cause of disability [1]. Up to 88% of individuals suffering stroke experience hemiparesis with disorders of gait and balance; which may persist even in the chronic phase [2, 3]. Therefore, a crucial goal in the rehabilitation of patients affected by stroke is to restore their mobility in order to allow resumption of independent living and improved quality of life [4, 5]. Post-stroke gait suffer from reduced abilities due to balance, speed, symmetry and muscle strength deficiencies [6]. Individuals suffering sub-acute and chronic stroke continue to use assistive or supportive devices in order to overcome postural control deficiencies, gait asymmetry, sensorimotor deficiencies and speed deterioration to increase their gait ability [7, 8]. Recent studies show that rehabilitation and gait training may also benefit chronic hemiplegic patients [9–11]. Involvement of a physical therapist is necessary to administer gait rehabilitation procedures, but due to the high incidence of stroke, the patient to therapist ratio is increasing and creating a situation which demands the increased use of engineering technology to aid therapists in gait rehabilitation interventions [12]. Gait rehabilitation after stroke requires intensive task-related training as well as variable training in changing environmental contexts with increasing physical demand [13]. Overground gait training with the use of assistive devices and in combination with other therapies or exercise regimens may benefit the gait rehabilitation after stroke [14].

Among the currently available gait rehabilitation systems, several recently developed devices offer gait training with overground walking for individuals suffering stroke [15–20]. These devices often use powered actuators for lower extremity joints, and may increase the post-stroke walking functionality towards a normal gait pattern. Meanwhile, the numerous limitations of the currently available systems such as high cost, wearability, weight, safety, and other issues often deny many individuals from undertaking post-stroke gait rehabilitation regimens with such devices. Therefore, it has been a difficult task to develop cost-efficient and adequate overground gait rehabilitation devices with a simple setup process and intuitive interactions targeted towards patients who can stand and move, but still need to overcome gait deficiencies.

Rehabilitation devices may also deliver sensory cues to the patients through visual, auditory and haptic modalities. These cues can be applied individually or in combination for task-oriented neuromotor rehabilitation following trauma [21]. Applications involving visual cues require a bulky setup (display monitor/surface). Therefore they are mostly not feasible for use with portable/wearable gait training devices. Auditory cues can be delivered using compact systems but are quite cumbersome for the users in gait training application, as they continuously occupy the hearing sensation and may obstruct activities of daily life. Moreover, noisy or inappropriately lit environments may also restrict the practical usability of auditory or visual cues [22]. The term haptic cue refers to the provision of information/experience to the users through their sense of touch. Visual and auditory senses are mostly occupied by receiving information to maintain the activities of daily life, whereas the haptic sensation is typically underutilized [23]. Thus, haptic cues may be delivered to the users without burdening their frequently used senses of vision and hearing. The ectoderm present at the early embryo stage is the basis of formation of both the skin and the nervous system [24]. This connection forms a link whereby the brain activity can be invoked through haptic stimuli, implying that the use of the haptic channel may provide an ease in changing neural plasticity. Haptic cues are generally divided into two categories; Tactile and Kinesthetic. Kinesthetic cues generally include a sensation of force at the location of the interface and offer a spatial frame of reference to the user. PHANTOM [25] and SPIDAR-G [26] are typical examples of devices that provide kinesthetic cues. Tactile Cues generally include the sensation of vibration, texture or pressure. These can be provided by devices such as LinkTouch [27] and MIMIC [28]. Thus, provision of haptic cues can be achieved through devices that deliver kinesthetic and tactile sensations to the user [29].

Provision of discrete kinesthetic cues to individuals with subacute stroke, through an instrumented cane, during overground walking has been found to improve paretic muscle activity [30]. Light touch (LT) refers to fingertip contact with another physical object [31]. When lightly touching a surface, proprioceptive receptors send information of position and velocity of the body sway to the cortical areas that control posture, leading to the activation of postural muscles to attenuate sway [32]. Continuous kinesthetic cues provided to individuals with chronic stroke through an instrumented haptic bar coupled with a visual display and with manipulation of physical environments improved their gait stability in challenging conditions such as downslope walking [33]. Tactile cues (muscle stimulation) provided to individuals suffering chronic stroke improved their gait performance during overground walking [34]. In addition to general physical therapy, tactile cues applied for therapy may improve gait performance in patients with chronic stroke and foot drop [35]. Thus, whether provided as kinesthetic or tactile, haptic cues may facilitate recovery of post-stroke gait during rehabilitation process. In this context, haptic cues can be a useful therapeutic option for intuitive interactions in overground gait rehabilitation; the applications of which in post-stroke ambulation recovery can be further explored.

We recently proposed a haptic cane device (HCD) which can deliver kinesthetic cue [36], and a vibrotactile feedback device (VFD) which can provide tactile cue [37]. Both HCD and VFD are low-cost and easy-to-use systems that can provide overground gait rehabilitation experience for individuals with sub-acute/chronic stroke. In the pilot study with HCD [36], we observed that proprioceptive augmentation delivered to individuals with sub-acute stroke could improve their paretic muscle activation and allow them to walk with increased gait speed on the ground while maintaining balance. Walking at increased gait speeds improves the kinematics and muscle activation patterns of hemiparetic gait [38], which is consistent with the speed-dependent changes reported in healthy subjects [39]. Increased gait speed also influences temporal symmetry of hemiparetic gait [40]. Moreover, post-stroke lower limb muscle functionality is correlated with comfortable and faster gait [41, 42]. Additionally in the pilot study with VFD [37], we observed that delivering tactile cue induces the increase in stance time of paretic lower limb, which effectively helps in reduction of temporal gait asymmetry. In studies related to upper limb, complementary integration of kinesthetic and tactile cues can improve hand movement perception [43], activate heteromodal areas to subserve multisensory integrative mechanisms at cortical and subcortical levels [44] and facilitate motor learning [45]. Novel rehabilitative approaches that combine simultaneous motor and sensory stimulations may substantially improve muscular strength and joint position sense in chronic stroke patients [46]; thereby confirming the strong impact of somatosensory stimulation on motor recovery [47]. The integration of kinesthetic and tactile cues for application to post-stroke gait rehabilitation has previously undergone little to no exploration [48]. Proprioceptive and tactile afferents both terminate and share overlapping networks in the somatosensory cortex [49]. Therefore, exploration of the effects of such cues on lower limb movements is warranted.

In this paper, we have explored the effects of combining the use of portable HCD and wearable VFD for a novel integrated cue system at various gait speeds. Our proposed system provides voluntary overground gait training modes for hemiparetic post-stroke ambulatory individuals. The first aim of this study is to determine whether the individual benefits of HCD and VFD can be observed as combined in the integrated cue system at user-preferred gait speed. The second aim of this study is to evaluate the effects of haptic cues (kinesthetic vs integrated) and increased gait speed on the characteristics of balance, gait symmetry and lower limb muscle activity.

## Methods

**Fig. 1:**
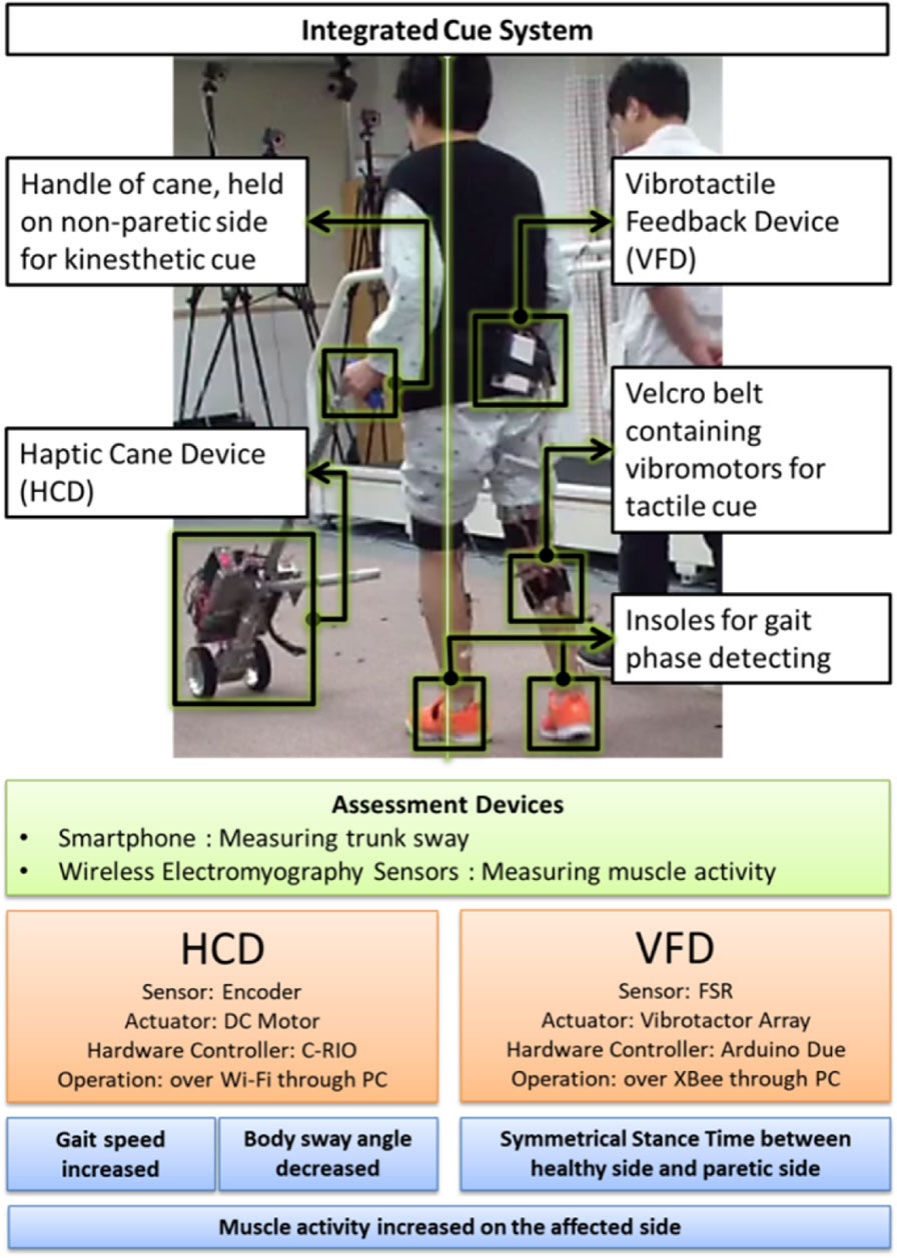
The proposed integrated cue system under use by a participant of this study. Participant is performing overground walk, whilst holding the handle of HCD by the healthy upper-limb and wearing the VFD meanwhile the vibrotactor array is positioned at the paretic shank. During all trials a physical therapist walks beside and monitors the participant

Integrated cue system with its components and application is shown in Fig. 1. The main idea of this system is to provide over-ground walking with aid of HCD for kinesthetic perception and a vibrotactor array attached on the paretic leg to cue the gait modification through VFD. Thus, integrated cue system has two basic functions, which are velocity control of the cane and vibration on the shank during swing phase of gait. The designs of HCD [36] and VFD [37] have individually been evaluated through pilot studies, the results of which have already been published.

HCD offers a new design, composed of a motorized wheel and a cane structure to provide continuous proprioceptive augmentation [36]. Canes and walkers have traditionally been used to improve the patient’s ability of walk by increasing the base of support area and also maintaining the voluntary gait by providing a psychological sense of stability [50]. However, these traditional devices require excessive use of the upper extremities to keep balance and manage weight distribution, which causes an aggravating gait imbalance due to the reduced involvement of the lower extremities. HCD prevents the patient to use excessive support force of an upper limb as a conventional cane and encourages the patient to use paretic lower limb more actively due to the reduced weight-bearing. The structure of HCD, which consists of a wheeled base and a cane, induces the balance stability by providing a continuous proprioceptive input and increasing user’s base of support. The HCD is velocity-controlled and can therefore help regulate the user’s gait speed. This makes it a valuable tool of gait rehabilitation in post-stroke ambulatory subjects. Use of the healthy hand is recommended for hemiparetic stroke subjects to operate and walk with HCD. As the users cannot apply excessive force for the weight support on the cane with a wheeled structure, they need to use their affected lower limb instead of chiefly relying on the upper limb. This operation mode of HCD provides a user with device-driven walking over the ground similar to treadmill-driven walking on a treadmill [51].

VFD is a wearable device which provides tactile cues to the user during overground walking and serves as a tool for diagnosing temporal asymmetry of gait. An elastic belt containing an array of six vibrotactors, securely attached to the inside of the belt, is used for the tactile cue application to the subject [37]. The vibrotactors cover the entire proximal end of paretic shank from front to back. Vibration with constant intensity at 200 Hz is provided during the paretic swing phase to maximally stimulate high-frequency Pacinian mechanoreceptors [52]. Each vibrotactor operates at 3.3 V and 66 mA, producing vibration amplitude of 1.4 G. The belt is worn on the lower leg, so that it does not interfere with proprioceptor information from the ground, as reduced feedback due to proprioceptive loss is likely to impair balance [53]. Also, direct muscle stimulation may contribute in enhancing the gait modification through afferent signal of vibration [54, 55]. The on/off status of the vibration signal can be visualized using an LED connected to the array of vibrotactors. The whole system can be worn easily using elastic belts with Velcro fasteners. A pair of insoles containing four force-sensitive resistors each, positioned at the heel, toe, fifth metatarsal, and first metatarsal are incorporated in VFD system, for collection of ground contact data.

### Subjects

To determine the effects of the proposed integrated cue system an experimental setup of overground walking was arranged. Ten individuals with hemiparetic stroke took part in the trials. Participants of the study had suffered single onset of unilateral hemiparetic stroke, and were in the sub-acute phase of recovery, were able to walk 10 m without assistance and had a 3 or higher Brunnstrom stage [56]. Individuals who were unable to follow verbal request, or had foot drop condition, limitations in joint range of motion, pain in the lower extremities, unstable medical conditions, or other diagnosed neurologic or musculoskeletal diseases were excluded from the study. Demographic details (mean ± standard deviation (SD) or counts) of the participants are presented in Table 1. All participants were inpatients of the Rehabilitation Center of Gyeongsang National University Hospital (Jinju, Republic of Korea). Gait training, strengthening and endurance exercise, and balance training were applied to the patients in their current rehabilitation program. All recruited subjects gave written informed consent approved by our local Ethics Committee before participating in the study.

**Table 1:** Demographic details of participants

Participants	10
Age (year)	57.7 ± 10.6
Height (cm)	165.5 ± 7.3
Weight (kg)	61.8 ± 10.1
Days since onset	62.5 ± 26.6
Gender	Male = 6, Female = 4
Cause of Stroke	Infarction = 5, Intracerebral Hemorrhage =5
Side of Hemiplegia	Right = 7, Left = 3

Values are mentioned as Mean ± SD or counts (as appropriate)

### Protocol

Each subject was first introduced to the system and its functionality. The patient grasps the handle of HCD to switch on its operation. HCD allows the patient to receive kinesthetic cue and walk within a range of gait speeds set by the operator. Meanwhile VFD provides a tactile cue on the paretic shank during swing phase. Subject wears a waist mounted leather belt holding a smartphone for evaluation of trunk sway and wireless electromyography (EMG) sensors (Wireless EMG Probes by NORAXON USA) [57] to monitor muscle activity. Recently, we utilized smartphone as a reliable tool to assess body sway parameters [48], [58, 59]. The smartphone ran a custom-made android application that identified trunk tilt and provided this information to a socket program on the operator’s computer at 100 Hz. EMG data was recorded at 1.5 kHz, for four muscles each of the paretic and healthy legs (vastus medialis obliquus (VMO), semitendinosus (SMT), tibialis anterior (TBA) and gastrocnemius medialis (GCM)). Stance times of healthy and paretic leg calculated by the Arduino Due on the VFD were sent to a custom-built MATLAB GUI through XBee. All subjects walked 10 m distance in each trial; data obtained during the middle 6.5 m was recorded for analysis. A 10 m walk can give an adequate representation of the post-stroke walking ability [60]. The scheme of administering haptic cues used in this study was adopted to assess their individual and combined effects on gait parameters. Furthermore, the speed of HCD was selected to achieve an increase of up to 40% in the user’s preferred gait speed. As, various exercise therapy regimes have been found to achieve up to 40% increase in initial gait speeds [61]. Each trial condition was performed two times by the participants and the mean was analyzed. Four cue combinations and three gait speeds were tested per participant. The cue combinations were No Cue walk (NCW), Tactile Cue walk (TCW), Kinesthetic Cue Walk (KCW), and Integrated Cue Walk (ICW). The speed settings were 0% (+ 0%), 20% (+ 20%), and 40% (+ 40%) increases in the subject’s normal gait speed. The details of the trial conditions are described in Table 2. All participants wore comfortable walking shoes with removable insoles to accommodate the custom-made insoles of the VFD. A physical therapist walked beside the subject during the walking trials. Participants were given a break of 1 min in between trials.

**Table 2:** Protocol of experimental trials

Trial	Conditions
No Cue Walk (NCW)	Normal walk in a straight line without any assistance/cue Subject maintains self-preferred walking speed Operator calculates the normal gait speed of the subject
Tactile Cue Walk (TCW)	Walk with only VFD’s tactile cue Subject maintains self-preferred walking speed Operator calculates the gait speed
Kinesthetic Cue Walk (KCW + 0%, KCW + 20%, KCW + 40%)	Walk with only HCD’s kinesthetic cue Speed is set to normal gait speed + 0%, + 20% and + 40% in separate trials
Integrated Cue Walk (ICW + 0%, ICW + 20%, ICW + 40%)	Walk with both VFD’s tactile cue and HCD’s kinesthetic cue Speed is set to normal gait speed + 0%, + 20% and + 40% in separate trials

Two trials per condition were performed with a break of 1 min between iterations

### Data analysis

**Fig. 2:**
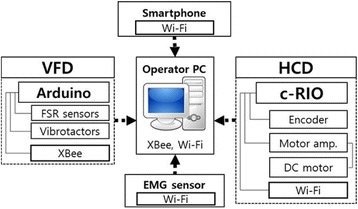
Block diagram of device communication. The individual devices (HCD, VFD, the smartphone and the wireless EMG recording sensors) were connected to the PC via wireless communication channels

During each walking trial, various parameters were recorded for post-experimental data analysis. Figure 2 illustrates the communication interface of the various devices used in this research. HCD was operated over Wi-Fi with a personal computer running a custom-built LabVIEW GUI program. This GUI commanded the HCD to setup various walking speeds during trials. It also handled the smartphone communication over Wi-Fi and saved the body sway data for post-experimental analysis. Later we calculated RMS of Mediolateral (ML) tilt to determine the condition of balance during various walking trials. The tilt in Mediolateral plane (ML tilt) is the sideways sway a human body experiences while maintaining an upright stance or walking. The RMS of ML Tilt has been utilized as a reliable marker of postural control during walking [62, 63]. Stance times received from the VFD were stored on the personal computer and utilized to define Stance Symmetry Ratio (i.e., Stance Symmetry Ratio (SSR) = stance time of healthy side / stance time of paretic side). The use of wireless EMG Sensors minimized the noise and artifacts of electrical and mechanical origins. The recorded EMG signals were band pass filtered (20–400 Hz), rectified, and smoothed with RMS filter at 100 ms [64]. To identify the increase in muscle activity of paretic side percentage of non-paretic peak activity (%NPA) was calculated, where integrals of EMG values of the paretic muscles during stance and swing phase were normalized to the EMG integrals from peak activity of the same muscles on the non-paretic side [30, 65, 66].

A one-way repeated measures analysis of variance (ANOVA) was performed to study the effects of Cue (factor), at preferred gait speed, which had four levels (no cue (NCW), tactile cue (TCW), kinesthetic cue (KCW + 0%) and integrated cue (ICW + 0%)) on RMS of ML Tilt, Stance Symmetry Ratio and %NPA in the four paretic muscles. Moreover, a two-way repeated measures ANOVA was used to identify the effects of Haptic Cue (factor, levels: kinesthetic (KCW) and integrated (ICW)) and increase in Gait Speed (factor, levels: 0%, 20% and 40%) on RMS of ML Tilt, Stance Symmetry Ratio and %NPA in the four paretic muscles. In addition, Mauchly’s test of Sphericity was used and Greenhouse-Geisser corrections were applied in case of its violation. Post hoc tests were conducted using the Bonferroni correction method. Partial eta squared (η^2^) was calculated as a measure of the effect size for one- and two-way repeated measure analysis of variance; Cohen’s d was also calculated and used for pairwise comparisons. All statistical analysis was performed using SPSS V20.0 (IBM Corp., Armonk, NY, USA).

## Results

**Fig. 3:**
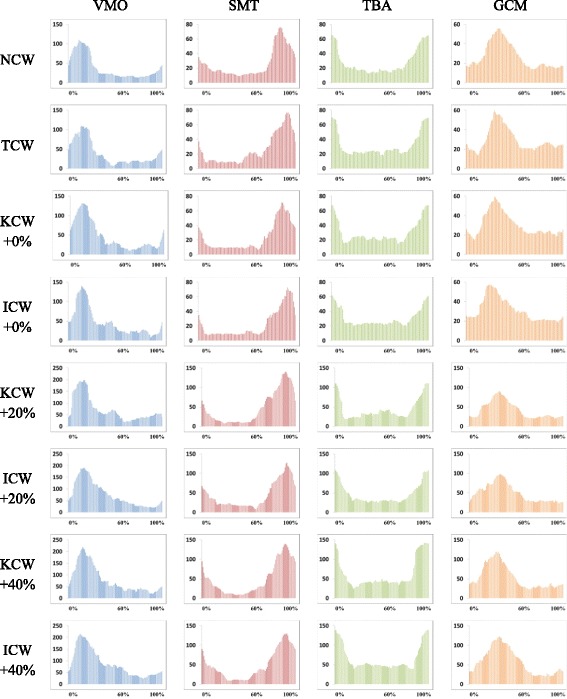
EMG activity profiles of a representative subject’s paretic muscles during trials. Paretic muscle activity profiles of a representative subject were calculated from the mean of 5 gait cycles. Here, the horizontal axis represents one full gait cycle beginning from stance phase of paretic leg and the vertical axis represents filtered EMG activity signal values in microvolts

Gait speed was calculated during NCW walking trial and was regulated with the HCD during KCW and ICW trials. It was an imposed parameter, except in the TCW trials, and all other measures were observed to evaluate the effects of haptic cues on the subjects. During TCW trials patients regulated the gait speed according to their comfort. Gait speed of the participants (Mean ± SD) during various walking trials is shown in Table 3. Descriptive statistics of the other measured parameters (Mean ± SD) from the post-experiment data analysis are also presented in Table 3. Figure 3 shows the activation profiles of the studied paretic muscles under different walking conditions of a representative subject. Furthermore, outputs of one-way ANOVA and two-way ANOVA are presented in Table 4 and Table 5, respectively. All presented outcomes of the multiple comparison post-hoc tests are obtained after application of Bonferroni correction. No post-hoc analysis was performed for the parameters that did not show any statistical significance in main effect and/or interaction.

**Table 3:** Details of the observed parameters during various walking trials

Trial Parameter	NCW	TCW	KCW + 0%	ICW + 0%	KCW + 20%	ICW + 20%	KCW + 40%	ICW + 40%
Gait Speed (m/s)	0.456 ± 0.164	0.485 ± 0.174	0.456 ± 0.164	0.456 ± 0.164	0.546 ± 0.196	0.546 ± 0.196	0.638 ± 0.229	0.638 ± 0.229
RMS of ML Tilt (degrees)	4.614 ± 1.085	4.530 ± 1.058	4.385 ± 1.223	4.482 ± 1.133	4.600 ± 1.003	4.676 ± 1.290	4.698 ± 1.110	4.775 ± 1.081
Stance Symmetry Ratio	1.109 ± 0.030	1.076 ± 0.023	1.101 ± 0.022	1.079 ± 0.024	1.078 ± 0.021	1.076 ± 0.020	1.096 ± 0.012	1.092 ± 0.015
%NPA of Paretic VMO in Stance	19.693 ± 3.238	20.867 ± 2.215	29.461 ± 1.979	30.067 ± 2.484	34.622 ± 1.699	34.969 ± 2.084	35.689 ± 1.619	35.536 ± 1.740
%NPA of Paretic VMO in Swing	10.950 ± 0.875	11.061 ± 0.541	11.373 ± 0.674	11.665 ± 0.787	11.657 ± 0.732	11.643 ± 0.675	11.586 ± 0.544	11.752 ± 0.651
%NPA of Paretic SMT in Stance	13.495 ± 1.058	13.614 ± 1.307	15.029 ± 1.274	15.293 ± 1.172	16.575 ± 0.830	16.229 ± 0.674	16.589 ± 0.818	16.559 ± 1.023
%NPA of Paretic SMT in Swing	7.641 ± 0.393	7.618 ± 0.366	7.803 ± 0.309	7.909 ± 0.290	7.951 ± 0.167	7.895 ± 0.255	7.998 ± 0.249	8.051 ± 0.245
%NPA of Paretic TBA in Stance	15.410 ± 1.496	15.272 ± 1.879	15.331 ± 1.614	15.815 ± 1.422	15.949 ± 0.953	16.235 ± 1.025	16.194 ± 1.849	16.173 ± 1.449
%NPA of Paretic TBA in Swing	4.765 ± 0.401	4.726 ± 0.405	4.738 ± 0.328	4.777 ± 0.414	4.853 ± 0.211	4.929 ± 0.309	4.869 ± 0.376	4.851 ± 0.387
%NPA of Paretic GCM in Stance	15.317 ± 1.584	14.852 ± 1.872	15.915 ± 1.135	15.379 ± 1.440	15.676 ± 1.413	15.462 ± 1.778	16.469 ± 0.909	16.484 ± 1.079
%NPA of Paretic GCM in Swing	4.726 ± 0.505	4.810 ± 0.374	4.662 ± 0.391	4.780 ± 0.325	4.857 ± 0.469	4.827 ± 0.309	4.824 ± 0.292	4.894 ± 0.539

The values for these parameters were recorded during each trial for each participant and are tabulated as Mean ± SD. A decrease in RMS of ML tilt relative to NCW indicated reduced trunk sway. Fall in stance symmetry ratio relative to NCW indicates improved temporal gait symmetry. Increase in %NPA is an indication of improved paretic muscle activity

**Table 4:** Results of one-way repeated measures ANOVA

Parameter	F	*P*-value	partial eta squared
RMS of ML Tilt	(3, 27) = 0.414	0.745	0.044
Stance Symmetry Ratio	(3, 27) = 21.204	**< 0.001**	**0.702**
%NPA of Paretic VMO in Stance	(3, 27) = 146.095	**< 0.001**	**0.954**
%NPA of Paretic VMO in Swing	(3, 27) = 2.752	0.062	0.022
%NPA of Paretic SMT in Stance	(1.683, 15.151) = 19.371	**< 0.001**	**0.793**
%NPA of Paretic SMT in Swing	(3, 27) = 2.537	0.078	0.220
%NPA of Paretic TBA in Stance	(3, 27) = 0.380	0.768	0.085
%NPA of Paretic TBA in Swing	(3, 27) = 0.059	0.981	0.006
%NPA of Paretic GCM in Stance	(3, 27) = 1.431	0.255	0.137
%NPA of Paretic GCM in Swing	(3, 27) = 0.620	0.608	0.058

Statically significant p-value and subsequent effect size are indicated in bold

**Table 5:** Results of two-way repeated measures ANOVA

Parameter	Factor	F	*P*-value	partial eta squared
RMS of ML Tilt	Haptic Cue	(1, 9) = 0.484	0.504	0.051
	Gait Speed	(1.253, 11.277) = 1.511	0.247	0.144
	Interaction	(2, 18) = 0.003	0.997	< 0.001
Stance Symmetry Ratio	Haptic Cue	(1, 9) = 14.448	**0.004**	**0.611**
	Gait Speed	(2, 18) = 11.786	**0.001**	**0.572**
	Interaction	(2, 18) = 9.988	**0.001**	**0.529**
%NPA of Paretic VMO in Stance	Haptic Cue	(1, 9) = 3.627	0.089	0.287
	Gait Speed	(2, 18) = 66.556	**< 0.001**	**0.881**
	Interaction	(2, 18) = 0.575	0.572	0.060
%NPA of Paretic VMO in Swing	Haptic Cue	(1, 9) = 0.741	0.412	0.076
	Gait Speed	(2, 18) = 0.359	0.703	0.038
	Interaction	(1.275, 11.477) = 0.655	0.471	0.068
%NPA of Paretic SMT in Stance	Haptic Cue	(1, 9) = 0.045	0.836	0.005
	Gait Speed	(1.208, 10.875) = 5.968	**0.028**	**0.399**
	Interaction	(2, 18) = 1.269	0.305	0.124
%NPA of Paretic SMT in Swing	Haptic Cue	(1, 9) = 0.548	0.478	0.057
	Gait Speed	(2, 18) = 1.643	0.221	0.154
	Interaction	(2, 18) = 1.905	0.178	0.175
%NPA of Paretic TBA in Stance	Haptic Cue	(1, 9) = 0.909	0.365	0.092
	Gait Speed	(2, 18) = 0.829	0.453	0.084
	Interaction	(2, 18) = 0.231	0.796	0.025
%NPA of Paretic TBA in Swing	Haptic Cue	(1, 9) = 0.323	0.584	0.035
	Gait Speed	(2, 18) = 1.848	0.186	0.170
	Interaction	(2, 18) = 0.608	0.934	0.008
%NPA of Paretic GCM in Stance	Haptic Cue	(1, 9) = 0.673	0.433	0.070
	Gait Speed	(2, 18) = 2.234	0.136	0.199
	Interaction	(2, 18) = 0.205	0.816	0.022
%NPA of Paretic GCM in Swing	Haptic Cue	(1, 9) = 0.387	0.549	0.041
	Gait Speed	(2, 18) = 1.370	0.276	0.132
	Interaction	(2, 18) = 0.284	0.756	0.031

Statically significant p-value and subsequent effect size are indicated in bold

**Fig. 4:**
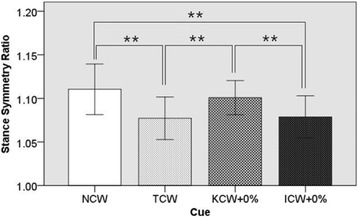
One-way repeated measures ANOVA of Stance Symmetry Ratio. Stance Symmetry Ratio shows statistically significant variations among different modes of haptic cues at preferred gait speed of the participants. Here: ** ⇒ *P* ≤ 0.01

Post hoc tests using the Bonferroni correction revealed (see Fig. 4) that the SSR was significantly lower in ICW + 0% condition than NCW (*P*-value = 0.003, Cohen’s d = 1.18) and KCW + 0% (P-value = 0.009, Cohen’s d = 1.00) conditions. Likewise, SSR was significantly lower in TCW condition than NCW (P-value = 0.002, Cohen’s d = 1.23) and KCW + 0% (P-value = 0.011, Cohen’s d = 1.06) conditions. However, no significant difference was found between NCW and KCW + 0%, and TCW and ICW + 0%.

**Fig. 5:**
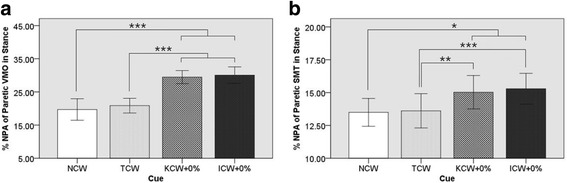
One-way repeated measures ANOVA of Precentage of Non-Paretic Peak Activitiy. (**a**) %NPA of Paretic VMO in Stance was significantly higher in ICW + 0% and KCW + 0% trials. (**b**) %NPA of Paretic SMT in Stance was significantly higher in ICW + 0% and KCW + 0% trials. Here: * ⇒ *P* ≤ 0.05, ** ⇒ P ≤ 0.01, *** ⇒ *P* ≤ 0.001

Moreover, post hoc tests also revealed (see Fig. 5. (a)) that the %NPA of Paretic VMO in Stance was significantly higher in ICW + 0% condition than NCW (*P*-value < 0.001, Cohen’s d = 3.59) and TCW (P-value < 0.001, Cohen’s d = 4.08) conditions. Likewise, it was significantly higher in KCW + 0% condition than NCW (P-value < 0.001, Cohen’s d = 3.64) and TCW (P-value < 0.001, Cohen’s d = 4.09) conditions. However, no significant difference was found between NCW and TCW, and KCW + 0% and ICW + 0%. Similarly, post hoc tests using the Bonferroni correction revealed (see Fig. 5. (b)) that the %NPA of Paretic SMT in Stance was significantly higher in ICW + 0% condition than NCW (*P*-value = 0.003, Cohen’s d = 1.61) and TCW (P-value < 0.001, Cohen’s d = 1.35) conditions. Likewise, it was significantly higher in KCW + 0% condition than NCW (*P*-value = 0.032, Cohen’s d = 1.31) and TCW (P-value = 0.001, Cohen’s d = 1.09) conditions. However, no significant difference was found between NCW and TCW, and KCW + 0% and ICW + 0%.

**Fig. 6:**
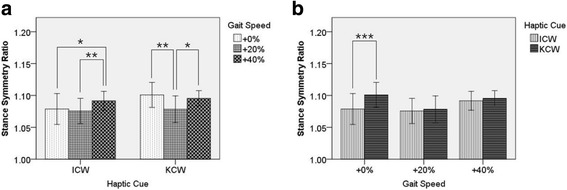
Two-way repeated measures ANOVA of Stance Symmetry Ratio. Due to significant interaction simple main effects were obesereved for post-hoc analysis. (**a**) Increase in gait speed from 0% to 20% exhibited improvements in SSR and further increase of gait speed to 40% worsened it. (**b**) Statistically significant improvement in SSR was found between KCW and ICW at 0% increased gait speed only. Here: * ⇒ P ≤ 0.05, ** ⇒ P ≤ 0.01, *** ⇒ P ≤ 0.001

Simple main effects were tested for post-hoc analysis due to the significant interaction of Haptic Cue and Gait Speed on Stance Symmetry Ratio. During ICW trials, significant difference was found between ICW + 0% and ICW + 40% (*P*-value = 0.047, Cohen’s d = 0.13), and ICW + 20% and ICW + 40% (P-value = 0.002, Cohen’s d = 0.09); suggesting increase in gait speed from 0% to 20% did not deliver any significant improvements and increase of gait speed to 40% slightly worsened the SSR (see Fig. 6. (a)). During KCW trials, significant difference was found between KCW + 0% and KCW + 20% (P-value = 0.003, Cohen’s d = 1.10), and KCW + 20% and KCW + 40% (*P*-value = 0.047, Cohen’s d = 0.99); suggesting increase in gait speed from 0% to 20% did deliver significant improvements but further increase of gait speed to 40% worsened the SSR (see Fig. 6. (a)). A statistically significant improvement in SSR was observed between KCW and ICW at 0% increased gait speed (*P*-value = 0.001, Cohen’s d = 1.00) (see Fig. 6. (b)) but it was not statistically significant at higher gait speeds (20% and 40%). In Summary, application of tactile cue at preferred gait speed (0%) shows improvements in SSR; increase in gait speed up to 20% using KCW also influences improvement in SSR but further increase is not beneficial.

**Fig. 7:**
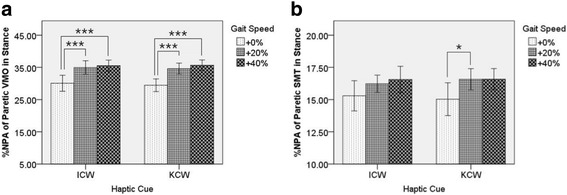
Two-way repeated measures ANOVA of Precentage of Non-Paretic Peak Activitiy. (**a**) Statistically significant increase of %NPA of Paretic VMO in Stance was found at higher gait speeds. (**b**) Statistically significant increase of %NPA of Paretic SMT in Stance existed between KCW + 0% and KCW + 20% only. Here: * ⇒ P ≤ 0.05, ** ⇒ P ≤ 0.01, *** ⇒ P ≤ 0.001

No significant interaction between Haptic Cue and Gait Speed on %NPA of Paretic VMO in Stance led to post-hoc analysis of Haptic Cue (ICW and KCW) separately (see Fig. 7. (a)). In ICW trials, significant difference was found between ICW + 0% and ICW + 20% (*P*-value = 0.001, Cohen’s d = 2.13), and ICW + 0% and ICW + 40% (P-value < 0.001, Cohen’s d = 2.55). While, no statistically significant difference existed between ICW + 20% and ICW + 40%. Similarly, during KCW trials, significant difference was found between KCW + 0% and KCW + 20% (P-value < 0.001, Cohen’s d = 2.79), and KCW + 0% and KCW + 40% (*P*-value < 0.001, Cohen’s d = 3.44). However, no significant difference existed between KCW + 20% and KCW + 40%. Also, no significant interaction between Haptic Cue and Gait Speed on %NPA of Paretic SMT in Stance led to post-hoc analysis of Haptic Cue (KCW and ICW) separately (see fig. 7. (b)). Significant improvement of muscle activity was found between KCW + 0% and KCW + 20% (*P*-value = 0.029, Cohen’s d = 1.43) only.

## Discussion

Our proposed system provides the user with an experience of over-ground walking. The overloading of upper limb is restricted by the design of haptic cane device (HCD) which increases involvement of lower limb paretic muscles. Contact of the user with the handle of HCD provides a continuous proprioceptive input; it serves the purpose of assisting balance. Meanwhile, the tactile cue provided by vibrotactile feedback device (VFD) enhances the gait modification through afferent signals of vibration. In this study we combined the promising prospects of kinesthetic and tactile haptic cues to comprise a novel integrated cue system.

### Effects of haptic cues delivered at preferred gait speed

The HCD allows the users to only apply limited load on the upper limb and encourages them to involve their paretic lower limb in order to achieve increase in muscle activity. In the No Cue Walk (NCW) and Tactile Cue Walk (TCW) trials, the participants possibly used compensatory and adaptive mechanisms of the nonparetic leg and trunk in motor control, where they coped with existing deficits including biomechanical changes rather than by regaining the appropriate sequence of muscle activation [67]. Whereas, with the introduction of the HCD in Kinesthetic Cue Walk (KCW) and Integrated Cue Walk (ICW) trials, the users tend to engage their paretic lower limb muscles more actively. Similar to our results, using an instrumented cane for restricted loading of upper limb led to increase in EMG of paretic leg muscles [30]. Vastus medialis obliquus (VMO) and semitendinosus (SMT) are the muscles vital in maintaining the upright body posture during stance phase of gait [32]. Thus, increased activation of these muscles at preferred gait speed also contributed to postural control during walking. Motor training to induce the use of the affected side increases activity in the lesioned hemisphere [68]. Therefore, the provision of a kinesthetic cue may facilitate the post-stroke gait rehabilitation by increasing paretic muscle activity.

Stroke disrupts the ability to maintain posture and equilibrium during walking [69]. With the use of HCD subjects may increase their base of support, thus allowing a greater range of center of mass motion while maintaining stability. Subject’s contact with the HCD also provides proprioceptive augmentation, which is beneficial in improving postural stability [30, 70]. Therefore, the contribution of kinesthetic cues from the hand to postural control suggests that the HCD may be useful not only in creating biomechanical advantages; but also in providing additional spatial orientation information for control of balance during overground walking. This is evident from the results obtained where the trunk sway (RMS of ML Tilt) of participants was better, albeit not statistically significantly so, during trials with HCD (KCW and ICW), as compared to those without it (NCW and TCW).

Hemiparesis is the most common impairment among the subjects who suffer a stroke, which can result in asymmetry of gait. In post-stroke gait, increase of the vertical ground reaction forces through the healthy limb is positively correlated with temporal asymmetry [71]. Therefore, musculoskeletal health of the non-paretic limb is also negatively affected by an asymmetric post-stroke gait. Stance symmetry ratio (SSR) is correlated with swing time and step length symmetry ratio, thus it can indicate improvements in gait symmetry [72]. A stance ratio of 1 is desired for normal/healthy walking condition [73]. Application of external cues using vibratory stimulation during gait may control gait parameters and improve gait performance in chronic stroke patients [34]. The afferent signals due to vibration increase the excitability of several segments of the spinal cord and could facilitate triggering of locomotor-like movements [74, 75]. High-frequency low-amplitude mechanical vibrations, applied to a muscle-tendon unit, generate afferent fiber discharges because of the activation of muscle spindles [74]. The modulation of afferent inputs alters the excitability of the corticospinal pathway [75] as well as the activation of cortical motor regions [76]. During low-amplitude biceps muscle tendon vibration, most of the functionally linked primary motor cortex cells respond mainly with excitatory firing [76]. At preferred gait speed during TCW trials, tactile cue induced substantial improvements in stance symmetry (as expected). This result may be due to increase of somatosensory afferent signals. Similarly, step-synchronized vibration stimulation of the soles improved gait steadiness in Parkinson’s disease patients with predominantly balance impairment, presumably by enhancing sensory feedback [77]. Proprioceptive afferents can play a key role in calibrating the spatial motor frame of reference and provide a powerful sensory augmentation to the central nervous system [78]. Moreover, gait speed and balance (Table 3) were not disturbed by provision of the tactile cues. Tactile cue is a constant vibration during swing phase to reduce the spasticity and improve the smoothness of motion in lower extremity during gait cycle, hence contributing towards improvement of gait symmetry. Temporal asymmetry is correlated with motor recovery [79]. Therefore, the provision of a tactile cue may facilitate post-stroke gait rehabilitation by improving the temporal stance symmetry.

The trunk sway of participants during walking as seen from the RMS of ML Tilt (Table 3) was similar in KCW + 0% and ICW + 0% but was better (not statistically significant) as compared to NCW trial. Increase in percentage of non-paretic peak activity (%NPA) of paretic muscles, VMO and SMT, was evident and alike in KCW + 0% and ICW + 0% as compared to NCW trial. Likewise, similar improvements of SSR in TCW and ICW + 0% trials were observed as compared to NCW trial. Thus at preferred gait speed, subjects tend to walk with increased symmetry and muscle activity due to the combined effects of HCD and VFD assimilating without disruption in balance.

### Effects of haptic cues delivered at increased gait speed

Gait speed is considered to be an important marker of deficit severity and functional ability after stroke [80]. Thus, speed gains resulting from the application of HCD’s kinesthetic cue may become the object of considerable interest in future clinical studies on gait rehabilitation. Post-stroke gait at increased speeds may allow more appropriate timing of lower limb muscles, improved movement coordination, and possibly facilitation of intra-limb and inter-limb energy transfers [81]. Therefore, the increase in muscle activity at higher gait speeds could be beneficial for post-stroke gait training. No statistically significant difference according to Haptic Cues in %NPA of paretic muscles existed as HCD was utilized in both conditions (ICW and KCW). On the other hand statistically significant difference in %NPA of paretic muscles, VMO and SMT, according to gait speed was observed, mainly attributed to the increase in gait speed.

We observed that participants could comfortably increase their gait speed while utilizing the proprioceptive augmentation through HCD. This proprioceptive augmentation aided the subjects in balance control during walking in KCW and ICW trials. No statistically significant difference at various walking speeds in RMS of ML tilt and no statistically significant difference according to Haptic Cues in RMS of ML tilt existed, as these trials included use of HCD.

Statistically significant improvement in SSR observed between KCW and ICW at 0% increased gait speed is attributed to the involvement of tactile cue. Whereas, no statistically significant difference in SSR at increased gait speeds (+ 20% and + 40%) was observed between KCW and ICW. This may be due to the increased influence of gait speed on temporal asymmetry as compared to that of tactile cue. As, post-stroke ambulatory subjects who are severely asymmetric appear more likely to exhibit improved temporal symmetry at their faster walking speeds [40]. For both ICW and KCW trials, increase in gait speed from 0% to 20% exhibited improvements in SSR and further increase of gait speed to 40% worsened it. This may be attributed to the limitations of hemiparetic gait. Level of gait functionality in post-stroke ambulatory subject limits the potential to increase the walking speed and introduces the need for compensations on the non-paretic side [82].

### Implications and future work

The suggested integrated cue system comprises of two independent devices (HCD and VFD) operated through a personal computer; however, for a patient self-usable system certain possible modifications are necessary which include the fusion of HCD and VFD into a single device, and making the device operable (data collection and monitoring) with a smartphone. The improvements in gait parameters, as observed during the use of integrated cues by the participants, including stance symmetry ratio and percentage of non-paretic peak activity will benefit the sub-acute unilateral hemiparetic stroke suffering individuals to overcome gait deficiencies. Nevertheless, it is essential to observe the translation of these improvements in clinical measures of gait rehabilitation (Functional Gait Assessment, 6 Minute Walk Test, Dynamic Gait Index, Timed Up and Go, etc.) following a gait training study using the proposed system. Observations with a small group of participants is a limitation of this study; thus further studies are necessary to explore the effects of the proposed system in detail with greater diversity of participants and grouping them into categories of different functional abilities and gait speeds. The future continuation of this research will include evaluation of the level of persistent gait improvement and long-term performance retention in participants. The eventual goal of this research is to devise a system that can be used in non-laboratory settings for gait rehabilitation of individuals with sub-acute stroke.

## Conclusions

The purpose of this paper was to identify the benefits of combining kinesthetic and tactile cues during overground walking, with preferred and increased gait speeds, delivered to individuals with unilateral hemiparetic stroke. The subjects could utilize the proposed integrated cue system at increased gait speeds, but the most significant benefits of the system were exhibited during user-preferred gait speed trials. At preferred gait speed, subjects tend to walk with increased temporal stance symmetry and paretic muscle activity without disruption in balance; thus indicating that combining the haptic cues was beneficial. Similar improvements were observed with 20% increase in the gait speed, where the improved muscle activity and gait symmetry more likely to have been induced by the increase in gait speed. On the other hand, those improvements faded away with further increase in gait speed due to the limitations of hemiparetic gait. This demonstrates that the efficacy of the proposed system is influenced by gait speed. In light of the above-mentioned observations, the integration of haptic cues may benefit post-stroke gait rehabilitation by inducing simultaneous improvement in gait symmetry and muscle activity.

## Abbreviations

%NPA: Percentage of non-paretic peak activity
ANOVA: Analysis of variance
EMG: Electromyography
GCM: Gastrocnemius medialis
HCD: Haptic cane device
ICW: Integrated cue walk
KCW: Kinesthetic cue walk
ML: Mediolateral
NCW: No cue walk
SD: Standard deviation
SMT: Semitendinosus
SSR: Stance symmetry ratio
TBA: Tibialis anterior
TCW: Tactile cue walk
VFD: Vibrotactile feedback device
VMO: Vastus medialis obliquus
η^2^: Eta squared

## References

[1] Adamson J, Beswick A, Ebrahim S (2004). Is stroke the most common cause of disability?. J Stroke Cerebrovasc Dis.

[2] Duncan PW, Zorowitz R, Bates B, Choi JY, Glasberg JJ, Graham GD, Katz RC, Lamberty K, Reker D (2005). Management of Adult Stroke Rehabilitation Care: a clinical practice guideline. Stroke.

[3] Winstein CJ, Stein J, Arena R, Bates B, Cherney LR, Cramer SC, Deruyter F, Eng JJ, Fisher B, Harvey RL, Lang CE, MacKay-Lyons M, Ottenbacher KJ, Pugh S, Reeves MJ, Richards LG, Stiers W, Zorowitz RD, American Heart Association Stroke Council, Council on Cardiovascular and Stroke Nursing, Council on Clinical Cardiology, Council on Quality of Care and Outcomes Research. Guidelines for Adult Stroke Rehabilitation and Recovery: A Guideline for Healthcare Professionals From the American Heart Association/American Stroke Association. Stroke. 2016;47(6):e98–169. 10.1161/STR.0000000000000098 27145936

[4] Langhammer B, Stanghelle JK, Lindmark B (2008). Exercise and health-related quality of life during the first year following acute stroke: a randomized controlled trial. Brain Inj.

[5] Duncan PW, Sullivan KJ, Behrman AL, Azen SP, Wu SS, Nadeau SE, Dobkin BH, Rose DK, Tilson JK (2007). Protocol for the locomotor experience applied post-stroke (LEAPS) trial: a randomized controlled trial. BMC Neurol.

[6] Eng JJ, Tang PF (2007). Gait training strategies to optimize walking ability in people with stroke: a synthesis of the evidence. Expert Rev Neurother.

[7] Earhart GM, Bastian AJ (2010). Evaluation of gait and turns. Handbook of Clin Neurophysiol.

[8] von Schroeder HP, Coutts RD, Lyden PD, Billings E Jr, Nickel VL: Gait parameters following stroke: a practical assessment**.** J Rehabil Res Dev 1995, 32(1): 25. 7760264

[9] Kwakkel G, Wagenaar RC, Twisk JW, Lankhorst GJ, Koetsier JC (1999). Intensity of leg and arm training after primary middle-cerebral-artery stroke: a randomised trial. Lancet.

[10] Byl NN, Pitsch EA, Abrams GM (2008). Functional outcomes can vary by dose: learning-based sensorimotor training for patients stable poststroke. Neurorehabil Neural Repair.

[11] Dias D, Lains J, Pereira A, Nunes R, Caldas J, Amaral C, Pires S, Costa A, Alves P, Moreira M, Garrido N (2007). Can we improve gait skills in chronic hemiplegics? A randomised control trial with gait trainer. Europa medicophysica.

[12] McHugh G, Swain ID (2013). A comparison between reported and ideal patient-to-therapist ratios for stroke rehabilitation. Health.

[13] Malouin FR, Richards CL. Assessment and training of locomotion after stroke: evolving concepts. In: Refshauge K, Ada L, Ellis E, editors. Science-based rehabilitation Theories into Practice. Edinburgh: Butterworth Heinemann; 2005. p. 185–222.

[14] Belda-Lois JM, Mena-del Horno S, Bermejo-Bosch I, Moreno JC, Pons JL, Farina D, Iosa M, Molinari M, Tamburella F, Ramos A, Caria A (2011). Rehabilitation of gait after stroke: a review towards a top-down approach. Journal of neuroengineering and rehabilitation.

[15] Veneman JF, Kruidhof R, Hekman EE, Ekkelenkamp R, Van Asseldonk EH, Van Der Kooij H (2007). Design and evaluation of the LOPES exoskeleton robot for interactive gait rehabilitation. IEEE Transactions on Neural Systems and Rehabilitation Engineering.

[16] Patton J, Brown DA, Peshkin M, Santos-Munné JJ, Makhlin A, Lewis E, Colgate EJ, Schwandt D (2008). KineAssist: design and development of a robotic overground gait and balance therapy device. Top Stroke Rehabil.

[17] Stauffer Y, Allemand Y, Bouri M, Fournier J, Clavel R, Métrailler P, Brodard R, Reynard F (2009). The WalkTrainer—a new generation of walking reeducation device combining orthoses and muscle stimulation. IEEE Transactions on Neural Systems and Rehabilitation Engineering.

[18] Luu TP, Low KH, Qu X, Lim HB, Hoon KH (2014). Hardware development and locomotion control strategy for an over-ground gait trainer: NaTUre-gaits. IEEE journal of translational engineering in health and medicine.

[19] Bortole M, Venkatakrishnan A, Zhu F, Moreno JC, Francisco GE, Pons JL, Contreras-Vidal JL (2015). The H2 robotic exoskeleton for gait rehabilitation after stroke: early findings from a clinical study. Journal of neuroengineering and rehabilitation.

[20] Nilsson A, Vreede KS, Häglund V, Kawamoto H, Sankai Y, Borg J (2014). Gait training early after stroke with a new exoskeleton–the hybrid assistive limb: a study of safety and feasibility. Journal of neuroengineering and rehabilitation.

[21] Huang H, Wolf SL, He J (2006). Recent developments in biofeedback for neuromotor rehabilitation. Journal of neuroengineering and rehabilitation.

[22] van Wegen E, de Goede C, Lim I, Rietberg M, Nieuwboer A, Willems A, Jones D, Rochester L, Hetherington V, Berendse H, Zijlmans J (2006). The effect of rhythmic somatosensory cueing on gait in patients with Parkinson's disease. J Neurol Sci.

[23] Zhang S, Wang D, Afzal N, Zhang Y, Wu R (2016). Rhythmic haptic stimuli improve short-term attention. IEEE transactions on haptics.

[24] Pispa J, Thesleff I (2003). Mechanisms of ectodermal organogenesis. Dev Biol.

[25] Massie TH, Salisbury JK (1994). The phantom haptic interface: a device for probing virtual objects. Proceedings of the ASME winter annual meeting, symposium on haptic interfaces for virtual environment and teleoperator systems.

[26] Kim S, Hasegawa S, Koike Y, Sato M. Tension based 7-DOF force feedback device: SPIDAR-G. Proceedings of IEEE Virtual Reality. 2002:283–4.

[27] Tsetserukou D, Hosokawa S, Terashima K. LinkTouch: a wearable haptic device with five-bar linkage mechanism for presentation of two-DOF force feedback at the fingerpad. Proceeding of the IEEE Haptics Symposium. 2014:307–12.

[28] Lee BC, Chen S, Sienko KH (2011). A wearable device for real-time motion error detection and vibrotactile instructional cuing. IEEE Transactions on Neural Systems and Rehabilitation Engineering.

[29] Ferber AR, Peshkin MA, Colgate JE (2009). Using kinesthetic and tactile cues to maintain exercise intensity. IEEE Trans Haptics.

[30] Boonsinsukh R, Panichareon L, Phansuwan-Pujito P (2009). Light touch cue through a cane improves pelvic stability during walking in stroke. Arch Phys Med Rehabil.

[31] Jeka JJ (1997). Light touch contact as a balance aid. Phys Ther.

[32] Winter DA, MacKinnon CD, Ruder GK, Wieman C (1993). An integrated EMG/biomechanical model of upper body balance and posture during human gait. Prog Brain Res.

[33] Fung J, Perez CF. Sensorimotor enhancement with a mixed reality system for balance and mobility rehabilitation. Proceedings of 33rd Annual International Conference of the IEEE Engineering in Medicine and Biology Society. 2011:6753–7. 10.1109/IEMBS.2011.6091666 22255889

[34] Park JM, Lim HS, Song CH (2015). The effect of external cues with vibratory stimulation on spatiotemporal gait parameters in chronic stroke patients. J Phys Ther Sci.

[35] Paoloni M, Mangone M, Scettri P, Procaccianti R, Cometa A, Santilli V (2010). Segmental muscle vibration improves walking in chronic stroke patients with foot drop: a randomized controlled trial. Neurorehabil Neural Repair.

[36] Pyo S, Oh MK, Yoon J. Development of an active haptic cane for gait rehabilitation. Proceedings of IEEE International Conference on Robotics and Automation. 2015:4464–9.

[37] Afzal MR, Oh MK, Lee CH, Park YS, Yoon J. A portable gait asymmetry rehabilitation system for individuals with stroke using a vibrotactile feedback. Biomed Res Int. 2015; 10.1155/2015/375638. 10.1155/2015/375638 PMC4486481 26161398

[38] Lamontagne A, Fung J (2004). Faster is better. Stroke.

[39] Murray MP, Mollinger LA, Gardner GM, Sepic SB (1984). Kinematic and EMG patterns during slow, free, and fast walking. J Orthop Res.

[40] Patterson KK, Parafianowicz I, Danells CJ, Closson V, Verrier MC, Staines WR, Black SE, McIlroy WE (2008). Gait asymmetry in community-ambulating stroke survivors. Arch Phys Med Rehabil.

[41] de Quervain IA, Simon SR, Leurgans SU, Pease WS, DA MALLISTER (1996). Gait pattern in the early recovery period after stroke. The Journal of Bone & Joint Surgery.

[42] Olney SJ, Griffin MP, Monga TN, McBride ID (1991). Work and power in gait of stroke patients. Arch Phys Med Rehabil.

[43] Blanchard C, Roll R, Roll JP, Kavounoudias A (2011). Combined contribution of tactile and proprioceptive feedback to hand movement perception. Brain Res.

[44] Kavounoudias A, Roll JP, Anton JL, Nazarian B, Roth M, Roll R (2008). Proprio-tactile integration for kinesthetic perception: an fMRI study. Neuropsychologia.

[45] Cuppone AV, Squeri V, Semprini M, Masia L, Konczak J (2016). Robot-assisted proprioceptive training with added vibro-tactile feedback enhances somatosensory and motor performance. PLoS One.

[46] Cordo P, Lutsep H, Cordo L, Wright WG, Cacciatore T, Skoss R (2009). Assisted movement with enhanced sensation (AMES): coupling motor and sensory to remediate motor deficits in chronic stroke patients. Neurorehabil Neural Repair.

[47] Conforto AB, Kaelin-Lang A, Cohen LG (2002). Increase in hand muscle strength of stroke patients after somatosensory stimulation. Ann Neurol.

[48] Afzal MR, Pyo S, Oh MK, Park YS, Lee BC, Yoon J. Haptic based gait rehabilitation system for stroke patients. Proceedings of IEEE/RSJ International Conference on Intelligent Robots and Systems. 2016:3198–203.

[49] Dijkerman C, De Haan E (2007). Somatosensory processes subserving perception and action. Behav Brain Sci.

[50] Van Hook FW, Demonbreun D, Weiss BD (2003). Ambulatory devices for chronic gait disorders in the elderly. Am Fam Physician.

[51] Yoon J, Park HS, Damiano DL (2012). A novel walking speed estimation scheme and its application to treadmill control for gait rehabilitation. Journal of neuroengineering and rehabilitation.

[52] Chatterjee A, Aggarwal V, Ramos A, Acharya S, Thakor NV (2007). A brain-computer interface with vibrotactile biofeedback for haptic information. Journal of NeuroEngineering and Rehabilitation.

[53] Pennycott A, Wyss D, Vallery H, Klamroth-Marganska V, Riener R (2012). Towards more effective robotic gait training for stroke rehabilitation: a review. Journal of neuroengineering and rehabilitation.

[54] Ivanenko YP, Grasso R, Lacquaniti F (2000). Influence of leg muscle vibration on human walking. J Neurophysiol.

[55] Gurfinkel VS, Levik YS, Kazennikov OV, Selionov VA (1998). Locomotor-like movements evoked by leg muscle vibration in humans. Eur J Neurosci.

[56] Brunnstrom S (1966). Motor testing procedures in hemiplegia: based on sequential recovery stages. Phys Ther.

[57] NORAXON Inc., TELEmyo DTS https://www.noraxon.com/noraxon-download/telemyo-dts-belt-receiver-user-manual/. Accessed 13 Apr 2018.

[58] Afzal MR, Byun HY, Oh MK, Yoon J (2015). Effects of kinesthetic haptic feedback on standing stability of young healthy subjects and stroke patients. Journal of neuroengineering and rehabilitation.

[59] Afzal MR, Oh MK, Choi HY, Yoon J (2016). A novel balance training system using multimodal biofeedback. Biomed Eng Online.

[60] Flansbjer UB, Holmbäck AM, Downham D, Patten C, Lexell J (2005). Reliability of gait performance tests in men and women with hemiparesis after stroke. J Rehabil Med.

[61] Dickstein R (2008). Rehabilitation of gait speed after stroke: a critical review of intervention approaches. Neurorehabil Neural Repair.

[62] Sienko KH, Balkwill MD, Oddsson LI, Wall C (2013). The effect of vibrotactile feedback on postural sway during locomotor activities. Journal of neuroengineering and rehabilitation.

[63] Wall C (2010). Application of vibrotactile feedback of body motion to improve rehabilitation in individuals with imbalance. Journal of neurologic physical therapy: JNPT.

[64] Krol P, Piecha M, Slomka K, Sobota G, Polak A, Juras G (2011). The effect of whole-body vibration frequency and amplitude on the myoelectric activity of vastus medialis and vastus lateralis. J sports sci med.

[65] Jung K, Kim Y, Cha Y, In TS, Hur YG, Chung Y (2015). Effects of gait training with a cane and an augmented pressure sensor for enhancement of weight bearing over the affected lower limb in patients with stroke: a randomized controlled pilot study. Clin Rehabil.

[66] Boonsinsukh R, Panichareon L, Saengsirisuwan V, Phansuwan-Pujito P (2011). Clinical identification for the use of light touch cues with a cane in gait rehabilitation poststroke. Top Stroke Rehabil.

[67] Buurke JH, Nene AV, Kwakkel G, Erren-Wolters V, IJzerman MJ, Hermens HJ (2008). Recovery of gait after stroke: what changes?. Neurorehabil Neural Repair.

[68] Richards LG, Stewart KC, Woodbury ML, Senesac C, Cauraugh JH (2008). Movement-dependent stroke recovery: a systematic review and meta-analysis of TMS and fMRI evidence. Neuropsychologia.

[69] Marigold DS, Eng JJ (2006). The relationship of asymmetric weight-bearing with postural sway and visual reliance in stroke. Gait & posture.

[70] Jeka JJ, Easton RD, Bentzen BL, Lackner JR (1996). Haptic cues for orientation and postural control. Attention, Perception, & Psychophysics.

[71] Kim CM, Eng JJ (2003). Symmetry in vertical ground reaction force is accompanied by symmetry in temporal but not distance variables of gait in persons with stroke. Gait & posture.

[72] Patterson KK, Gage WH, Brooks D, Black SE, McIlroy WE (2010). Evaluation of gait symmetry after stroke: a comparison of current methods and recommendations for standardization. Gait & posture.

[73] Patterson KK, Mansfield A, Biasin L, Brunton K, Inness EL, McIlroy WE (2015). Longitudinal changes in poststroke spatiotemporal gait asymmetry over inpatient rehabilitation. Neurorehabil Neural Repair.

[74] Roll JP, Vedel JP, Ribot E (1989). Alteration of proprioceptive messages induced by tendon vibration in man: a microneurographic study. Exp Brain Res.

[75] Steyvers M, Levin O, Van Baelen M, Swinnen SP (2003). Corticospinal excitability changes following prolonged muscle tendon vibration. Neuroreport.

[76] Fourment A, Chennevelle JM, Belhaj-Saif A, Maton B (1996). Responses of motor cortical cells to short trains of vibration. Exp Brain Res.

[77] Novak P, Novak V (2006). Effect of step-synchronized vibration stimulation of soles on gait in Parkinson's disease: a pilot study. Journal of neuroengineering and rehabilitation.

[78] Bard C, Fleury M, Teasdale N, Paillard J, Nougier V (1995). Contribution of proprioception for calibrating and updating the motor space. Can J Physiol Pharmacol.

[79] Hsu AL, Tang PF, Jan MH (2003). Analysis of impairments influencing gait velocity and asymmetry of hemiplegic patients after mild to moderate stroke. Arch Phys Med Rehabil.

[80] Patterson SL, Forrester LW, Rodgers MM, Ryan AS, Ivey FM, Sorkin JD, Macko RF (2007). Determinants of walking function after stroke: differences by deficit severity. Arch Phys Med Rehabil.

[81] Hesse S, Werner C, Paul T, Bardeleben A, Chaler J (2001). Influence of walking speed on lower limb muscle activity and energy consumption during treadmill walking of hemiparetic patients. Arch Phys Med Rehabil.

[82] Jonkers I, Delp S, Patten C (2009). Capacity to increase walking speed is limited by impaired hip and ankle power generation in lower functioning persons post-stroke. Gait & posture.

